# A sequencing strategy for identifying variation throughout the prion gene of BSE-affected cattle

**DOI:** 10.1186/1756-0500-1-32

**Published:** 2008-06-23

**Authors:** Michael L Clawson, Michael P Heaton, John W Keele, Timothy PL Smith, Gregory P Harhay, Juergen A Richt, William W Laegreid

**Affiliations:** 1 United States Department of Agriculture (USDA), Agricultural Research Service (ARS), US Meat Animal Research Center (USMARC), Clay Center, NE 68933, USA; 2 USDA, ARS, National Animal Disease Center, PO Box 70, Ames, IA 50010, USA; 3 University of Illinois at Champaign-Urbana, Department of Pathobiology, 2001 S. Lincoln Avenue, Urbana, IL 61802, USA

## Abstract

**Background:**

Classical and atypical bovine spongiform encephalopathies (BSEs) are cattle prion diseases. Distinct bovine prion gene (*PRNP*) alleles have been associated with classical and atypical BSE susceptibility. However, the full extent of *PRNP *allele association with BSE susceptibility is not known. A systematic sequence-based genotyping method that detects variation throughout *PRNP *would be useful for: 1) detecting rare *PRNP *alleles that may be present in BSE-affected animals and 2) testing *PRNP *alleles for an association with either classical or atypical BSE susceptibility.

**Findings:**

We improved a Sanger-based sequencing strategy for detecting bovine *PRNP *variation through all exons, introns, and part of the promoter (25.2 kb). Our current method can detect 389 known and other potentially unknown *PRNP *polymorphisms that may be present in BSE-affected cattle. We determined *PRNP *genotypes for the first U.S. BSE case and her sire. Previously unknown *PRNP *polymorphisms were not detected in either animal and all *PRNP *genotypes support the sire-daughter relationship.

**Conclusion:**

The methodologies described here characterize variation throughout *PRNP*. Consequently, rare *PRNP *alleles that may be present in BSE-affected cattle can be detected.

## Introduction

Transmissible spongiform encephalopathies (TSEs), also known as prion diseases, are a class of neurodegenerative disorders that occur in humans, ruminants, cats, and mink [1]. Three distinct TSEs afflict cattle: classical bovine spongiform encephalopathy (BSE), atypical H-type BSE, and atypical L-type BSE [2-4]. Bovine prion gene (*PRNP*) alleles are associated with classical and atypical BSE susceptibility [5-8]. However, the extent of bovine *PRNP *alleles that predict BSE susceptibility has not yet been determined.

Classical BSE is acquired by cattle through the consumption of meat and bone meal contaminated with the infectious prion agent [9]. Classical BSE susceptibility is associated with the deletion alleles of two bovine *PRNP *insertion/deletion polymorphisms, one within the promoter region and the other in intron 1 [5-7]. Both of these polymorphisms have alleles in linkage disequilibrium (LD) with the alleles of at least 43 other *PRNP *polymorphisms in *Bos taurus *cattle [10]. Thus, additional *PRNP *alleles are likely to associate with classical BSE susceptibility with comparable if not greater significance than the indel alleles.

Atypical BSEs have recently been identified in Asian, North American, and European cattle [11]. Two *PRNP *alleles are implicated with atypical BSE susceptibility. A non-synonymous polymorphism (E211K) was found by one of us (J.A.R.) within the prion coding region of an H-type atypical BSE case identified in the U.S. (2006). The E211K polymorphism is homologous to the human E200K polymorphism, a risk factor for genetic Creutzfeldt-Jakob disease [12], and is the suspected cause of the atypical BSE case. The 211 K allele is exceedingly rare and has not been found in other atypical BSE cases or healthy cattle [10,13,14]. However, a *PRNP *haplotype that is associated with atypical BSE susceptibility was found in H- and L-type atypical cases from Canada, France, and the U.S., and may have widespread involvement with atypical BSE [8]. The haplotype spans part of *PRNP *intron 2, the entire coding region of exon 3, and part of the three prime untranslated region of exon 3 (13 kb) [8,10]. Alleles that may be causative for atypical BSE susceptibility, including those that may be within *PRNP*, are thought to be linked with the implicated haplotype in atypical BSE cases [8].

Efforts to identify *PRNP *alleles that predict classical or atypical BSE susceptibility require improved methods to: 1) efficiently extract DNA from available tissue samples and 2) identify variation throughout *PRNP*. The brainstem obex is the specified sample for BSE testing [15]. If an obex sample is BSE positive and the carcass from which it originated is destroyed before additional samples are collected, the obex becomes the sole source of transmissible material and DNA from the BSE-afflicted animal. Thus, the ability to obtain DNA from obex tissue is a critical prerequisite for *PRNP *genotyping. The ability to accurately genotype known *PRNP *polymorphisms and identify additional *PRNP *polymorphisms that may be present in BSE-afflicted animals is also important. Here, we provide an efficient method of DNA extraction from bovine obex and report a Sanger-based sequencing strategy that comprehensively identifies variation throughout *PRNP *(25.2 kb). We determined the *PRNP *sequence and haplotypes of the U.S. case of classical BSE (2003) and her sire, show that both animals do not have previously unrecognized *PRNP *variation, and that their parent-offspring relationship is supported throughout *PRNP*.

### DNA isolation method

For DNA isolation, ten cattle obexes (0.7 – 1.9 grams) were individually homogenized in PBS (weight per volume = 25%). Three hundred and fifty microliters of the homogenate, containing 87.5 mg of obex, was digested overnight in proteinase K. One half of the digestion was frozen while the other half was subjected to standard Tris-saturated phenol:chloroform:isoamyl alcohol (25:24:1, pH 8) and chloroform extractions, followed by salt precipitation, ethanol washing, and re-suspension in TE (see additional file 1 for supplementary methods on DNA isolation from cattle obex tissue and quantification). The procedure yielded a mean of 12.1 μg DNA (high = 17.34 μg, low = 7.64 μg, Table 1). Each extraction yielded more than 3× the 2.4 μg of DNA typically used to comprehensively amplify and sequence *PRNP*. The DNAs were obtained from the equivalent of 43.75 mg of obex, or less than 7% of each obex. Thus, DNA sufficient for *PRNP *genotyping can be obtained from a small portion of the obex tissue.

**Table 1:** Cattle obex DNA yield and quality

Sample^a^	DNA Yield (μg)	DNA concentration (μg/mL)^b^	260/280 nm
1	15.55	155.54	1.87
2	17.34	173.39	1.87
3	10.78	107.82	1.87
4	13.34	133.39	1.84
5	7.80	77.97	1.83
6	7.64	76.40	1.83
7	17.81	178.07	1.86
8	9.76	97.55	1.80
9	10.09	100.86	1.83
10	10.70	106.98	1.84

^a^Ten cattle obex tissues (44 mg each).

^b^0.1 mL suspension.

Mean yield = 12.1 μg, SD = 3.7, 95% CI = ± 2.3.

### Development of oligonucleotides for *PRNP *amplification and sequence genotyping

**Figure 1:**
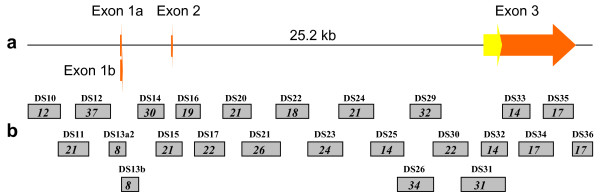
***PRNP *coverage**. **(a) Sequence-based physical map of bovine *PRNP***. Orange and yellow arrows represent untranslated and protein coding regions, respectively. **(b) *PRNP *amplicons**. Grey rectangles represent 24 overlapping amplicons. Numbers assigned to the amplicons are above the rectangles. Numbers of polymorphisms observed in U.S. cattle in genomic regions spanned by the amplicons are shown inside the rectangles.

**Figure 2:**
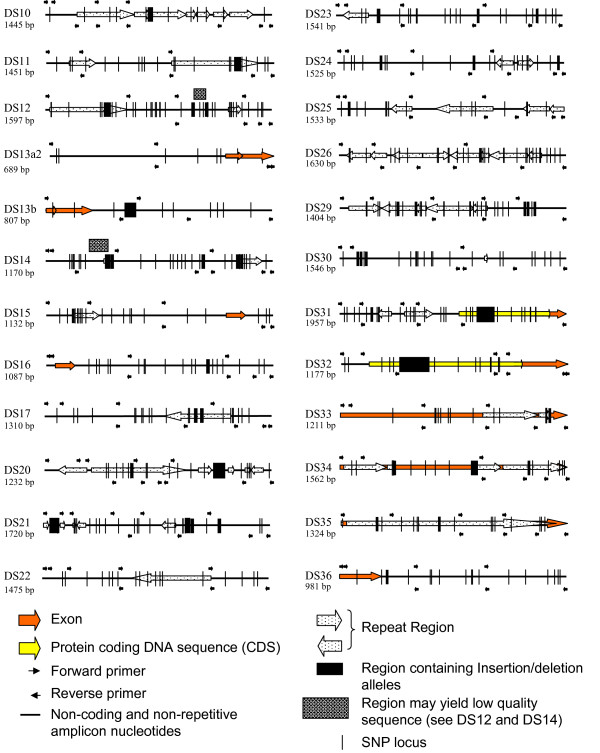
**Physical maps of the 24 BTAPRNP amplicons that collectively span bovine *PRNP *(25.2 kb)**. Alternate spicing of Exon 1 is annotated on DS13a2 and DS13b. Amplicon sizes may vary by the presence or absence of Insertion/Deletion alleles.

We improved a set of oligonucleotides that can be used to: 1) collectively amplify *PRNP *(25.2 kb) on 24 overlapping amplicons (Figure 1b) and 2) genotype *PRNP *variation by sequence [10]. This new set is comprised of 189 oligonucleotides, of which 168 have been previously described [10]. All of the oligonucleotides are designed around 389 known *PRNP *polymorphisms (48 amplification/sequencing, 141 sequencing; see additional file 2 for a table of oligonucleotides for *PRNP *amplification and sequencing). Ten *PRNP *amplicons described previously [10] have been modified with redesigned oligonucleotides due to unsatisfactory performance, including one that covers the InDel within intron 1 that is associated with classical BSE susceptibility and another that covers the entire coding region of exon 3. Strict PCR conditions and/or reagents for many of these amplicons are required (see additional file #3 for supplementary PCR information). All 189 oligonucleotides of the set have been tested for amplification and/or sequencing performance on 192 cattle DNAs representing 21 breeds (see additional file #3 for breed information) [10,16]. Three hundred and eighty-eight *PRNP *polymorphisms have been observed in these cattle DNAs [10]. None of the oligonucleotides hybridize to genomic sites containing the 388 polymorphisms or E211K (Figure 2).

Additional *PRNP *variation is predicted to exist in cattle and some of it could affect the hybridization of oligonucleotides within the set. This likelihood is accounted for by overlapping *PRNP *amplicons coupled with overlapping *PRNP *nucleotide coverage by sequencing oligonucleotides (Figure 2). Thus, the set will facilitate the detection of both known and unknown *PRNP *polymorphisms, including those within primer hybridization sites.

Complete PCR coverage of *PRNP *(25.2 kb) from a BSE-positive animal can be conducted in a single 96-well plate using a thermocycler capable of zoned heating cycles (see additional file #4 for a 96-well PCR design). Two 55-μL PCRs for each of the 24 *PRNP *amplicons yield enough template to sequence *PRNP *at least twice with each of the 189 oligonucleotides on an ABI capillary 3730 sequencer. This coverage can yield approximately eight-fold coverage of each *PRNP *nucleotide base throughout the 25.2-kb region, except for several small problematic regions (Figure 2). Each amplification reaction requires 50 ng of DNA. Consequently, thorough coverage of *PRNP *can be achieved with 2.4 μg of genomic DNA. All of the sequencing reactions required for duplicate coverage of *PRNP *can fit on a single 384-well plate.

### Complete *PRNP *sequences of the U.S. classical BSE case of 2003 and her sire

The oligonucleotide set was used to amplify and sequence *PRNP *from: 1) the U.S. case of classical BSE (2003), a Holstein cow imported from Canada, and 2) her Holstein sire. All sequence nucleotides were scored with Phred, aligned with Phrap, edited in Consed, and genotyped with Polyphred software [17]. Unambiguous genotypes for 388 *PRNP *polymorphisms were scored for the U.S. BSE case and her sire (see additional file #5 for a complete list of genotypes and GenBank files EU130450 and EU130451). Sequences from both animals did not reveal any previously unknown *PRNP *polymorphisms. Both animals are homozygous for the deletion alleles of the two InDels previously associated with classical BSE susceptibility. All of the *PRNP *polymorphism genotypes support the sire-offspring relationship.

### *PRNP *haplotypes of the BSE case and her sire

**Figure 3:**
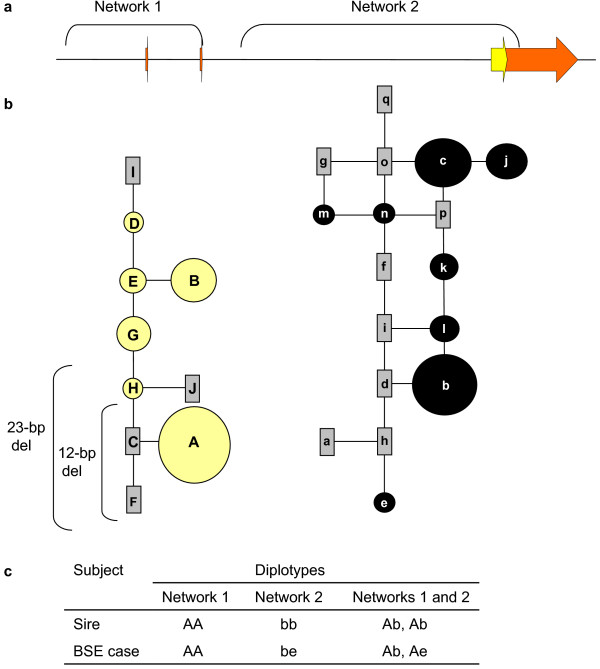
***PRNP *haplotypes of the U.S. classical BSE case and her sire**. **(a) Physical map of haplotype networks one and two that span *PRNP *regions of high and low LD, respectively**. **(b) *PRNP *haplotype relationships**. Circles represent *PRNP *haplotypes observed in a group of 86 U.S. Holsteins. Circle areas are proportional to haplotype frequencies. Rectangles represent haplotypes with a frequency of less than 2.5% in the Holstein group. Vertical brackets denote haplotypes with deletion alleles from a 23-bp and/or 12-bp InDel previously associated with classical BSE susceptibility. **(c) *PRNP *diplotypes of the U.S. classical BSE case and her sire**. The frequencies of diplotypes "Ab" and "Ae" in the Holstein group are 0.264 and 0.030, respectively.

Nineteen haplotype tagging polymorphisms (htSNPs) [10] were used to phase *PRNP *haplotypes of the classical BSE case and her sire (see additional file #6 for a description of the phasing method [18,19]). The htSNPs define *PRNP *haplotypes within two independent Median-Joining networks that account for high and low LD regions observed in *Bos taurus *cattle populations [10] (Figure 3, see additional file #7 for haplotype sequences). Nine htSNPs define haplotypes within a region of high LD that spans the *PRNP *promoter through a small portion of intron 2 (network one). Ten other htSNPs define haplotypes within a *PRNP *region of low LD that span most of intron 2 through the 3'UTR (network two). The htSNPs collectively define haplotype diversity within and across *PRNP *and have been previously used to test *PRNP *variation for an association with atypical BSE susceptibility [8].

*PRNP *haplotypes of the classical BSE case and her sire were compared to each other and to those from 86 U.S. Holsteins, of which 82 were part of the USMARC Dairy Cattle Panel (MDCP1.5) [10] and 4 were part of the USMARC Beef Cattle Discovery Panel 2.1 (MBCDP2.1) [16]. The classical BSE case and her sire were both homozygous for a haplotype in network one that is common in U.S. Holstein (haplotype A, Figure 3). The two InDel deletion alleles that associate with BSE susceptibility are present on this haplotype. However, while the sire was homozygous for a common *PRNP *haplotype in network two (haplotype b), the classical BSE case was heterozygous for the same haplotype and one much less common (haplotype e). The frequencies of haplotype combinations "Ab" and "Ae" in the Holstein DNAs are 0.264 and 0.030, respectively. The significance of these haplotype combinations and classical BSE susceptibility is unknown. *PRNP *haplotype combinations such as those described here can be tested for association with either classical or atypical BSEs in larger studies.

## Conclusion

BSE has grown in complexity with the identification of classical and atypical types. *PRNP *alleles associate with classical and atypical BSE susceptibility. The methodologies described here characterize variation throughout *PRNP*. Consequently, rare *PRNP *alleles that may be present in BSE-affected cattle can be detected.

## Competing interests

The authors declare that they have no competing interests.

## Authors' contributions

MLC conceived the project, developed DNA extraction procedures, designed and tested oligonucleotides, analyzed sequence, and wrote the manuscript. MPH designed oligonucleotides, developed DNA extraction procedures, and contributed DNA samples to the study. JWK assisted in sequence analyses. TPLS conducted sequencing and sequence analyses. GPH assisted in haplotype analyses. JAR contributed DNA samples to the study and improved oligonucleotide design. WWL designed oligonucleotides and assisted in sequence analyses. All authors read and approved the final manuscript.

## Supplementary Material

Additional file 1 Supplementary Methods – DNA isolation from cattle obex and quantification. Detailed technical and reagent information regarding DNA isolation from cattle obex tissue. Click here for file

Additional file 2 Supplementary Table – Oligonucleotides for *PRNP *amplification and DNA sequencing [USMARC Bovine *PRNP *Oligonucleotide Set Version 4 (MBPRNPOSv4)]. Complete list of oligonucleotides used for *PRNP *amplification and DNA sequencing. Click here for file

Additional file 3 Supplementary Methods – Cattle DNAs used for oligonucleotide testing, PCR reagents, and thermocycling conditions. Complete list of cattle breed DNAs used for oligonucleotide testing and technical information on PCR reagents and thermocycling conditions for *PRNP *amplification. Click here for file

Additional file 4 Supplementary Figure – *PRNP *PCRs in 96 well format. Illustration of *PRNP *amplification in a 96 well plate. Click here for file

Additional file 5 Supplementary Table – *PRNP *genotypes of the U.S. classical BSE case and her sire. Side-by-side comparison of *PRNP *genotypes of the U.S. classical BSE case and her sire. Click here for file

Additional file 6 Supplementary Methods – *PRNP *haplotype reconstructions. Method for determining *PRNP *haplotypes in the group of 86 Holsteins, the classical BSE case, and her sire. Click here for file

Additional file 7 Supplementary Table – *PRNP *haplotype sequences in Networks 1 and 2. *PRNP *haplotype sequences represented in Networks 1 and 2 of Figure 3. Note: This table was originally published in PLoS ONE [8]. Click here for file
