# Supplementary material for: A sequencing strategy for identifying variation throughout the prion gene of BSE-affected cattle
Source: BMC Res Notes. 2008 Jun 23;1:32. doi: 10.1186/1756-0500-1-32 (PMC2525647; doi:10.1186/1756-0500-1-32)
Supplement: Additional file 1 — Supplementary Methods – DNA isolation from cattle obex and quantification. Detailed technical and reagent information regarding DNA isolation from cattle obex tissue. [file 1756-0500-1-32-S1.doc]

**Supplementary Methods - DNA isolation from cattle obex tissue and quantification**

Ten obexes were collected from cattle immediately following slaughter at a Nebraska commercial facility and stored at 4ºC.

Each obex sample was suspended in PBS (weight per volume = 25%) and homogenized with a tissue grinder.

350 μL of the homogenate were placed in a 15-mL conical tube.

2.26 mL of Nuclei Freezing buffer [(NFB): 10 mM Tris-HCL, 400 mM NaCl, 2 mM EDTA, PH 8.0] with 1% SDS and 600 ng Rnase A were added to the homogenate.

The tube contents were mixed by shaking at 37ºC for 1 hr.

Proteinase K (1.25 mg) was added to the tube contents.

Tube contents were mixed by shaking at 37ºC overnight.

One half of the tube contents (1.3 mL) were transferred to an Eppendorf (Westbury, NY) 15 mL Phase Lock Gel^TM^ tube. The remaining half was frozen and stored.

Two organic extractions were done with 1.3 mL of Tris-saturated phenol:chloroform:isoamyl alcohol (25:24:1) pH 8.0.

One aqueous extraction was done with 1.3 mL chloroform.

Aqueous extracts were transferred to fresh 15-mL tubes.

125 μL 3 M NaOAC pH 5.2 and 2.75 mL of 100% ETOH were added to the 15-mL tubes followed by mixing.

The tubes were incubated at -80ºC for 1 hr.

The tubes were centrifuged at 2400 x g at 4ºC for 30 min (Beckman Coulter Allegra 6R Centrifuge, GH3.8 rotor, Fullerton, CA).

Pelleted DNA was washed with 2.5 mL of 70% ethanol (4ºC).

The tubes were re-centrifuged at 2400 x g at 4ºC for 10 min.

Tube fluids were decanted and the pellets were air dried at room temperature for 15 minutes.

DNA pellets were suspended in 100 μL of TE (10 mM Tris pH 7.5, 1 mM EDTA).

DNA absorption at 260 nm and 280 nm was determined with a NanoDrop Technologies ND-1000 spectrophotometer (Wilmington, DE).
