# Supplementary material for: A sequencing strategy for identifying variation throughout the prion gene of BSE-affected cattle
Source: BMC Res Notes. 2008 Jun 23;1:32. doi: 10.1186/1756-0500-1-32 (PMC2525647; doi:10.1186/1756-0500-1-32)
Supplement: Additional file 3 — Supplementary Methods – Cattle DNAs used for oligonucleotide testing, PCR reagents, and thermocycling conditions. Complete list of cattle breed DNAs used for oligonucleotide testing and technical information on PCR reagents and thermocycling conditions for PRNP amplification. [file 1756-0500-1-32-S3.doc]

**Supplementary Methods - Cattle breeds used for oligonucleotide testing, PCR reagents, and thermocycling conditions**

**192 cattle DNAs representing 21 breeds were used for oligonucleotide testing.**

The DNAs used for oligonucleotide testing comprise the U.S. Meat Animal Research Center (USMARC) Beef Cattle Discovery Panel 2.1 (MBCDP2.1) [16] and the USMARC Dairy Cattle Panel (MDCP1.5) [10]. The following breeds are represented by the two panels:

Angus (n = 8), Hereford (n = 8), Limousin (n = 8), Simmental (n = 7), Charolais (n = 6), Beefmaster (n = 5), Red Angus (n = 6), Gelbvieh (n = 6), Brangus (n = 5), Salers (n = 5), Brahman (n = 6), Shorthorn (n = 5), Maine-Anjou (n = 5), Longhorn (n = 4), St. Gertrudis (n = 4), Chianina (n = 4), Holstein (n = 86), Jersey (n = 7), Guernsey (n = 3), Aryshire (n = 2) and Brown Swiss (n = 2).

**PCR Reagents**

All 55 μL PCRs were conducted with 1.1X Thermo-Start PCR Master Mix (ABgene, United Kingdom).

PCRs were conducted in a Bio-Rad PTC 200 Peltier Thermal Cycler or a Bio-Rad Dyad Peltier Thermal Cycler (Hercules, CA).

Reagents for all bovine prion amplifications EXCEPT for DS13a2 and DS13b are the following:

Thermo-Start DNA Polymerase 1.25 units

MgCl_2_ 2.3 mM

dNTPs 0.181 mM

Forward Amplification Primer 0.4 μM

Reverse Amplification Primer 0.4 μM

Genomic DNA 50 ng

**Amplification reagents for bovine prion amplicon DS13a2.**

Thermo-Start DNA Polymerase 1.25 units

MgCl_2_ 1.36 mM

dNTPs 0.181 mM

Forward Amplification Primer 0.4 μM

Reverse Amplification Primer 0.4 μM

DMSO 3% final concentration

Genomic DNA 50 ng

**Amplification reagents for bovine prion amplicon DS13b.**

Thermo-Start DNA Polymerase 1.25 units

MgCl_2_ 1.36 mM

dNTPs 0.181 mM

Forward Amplification Primer 0.4 μM

Reverse Amplification Primer 0.4 μM

DMSO 2% final concentration

Genomic DNA 50 ng

**PCR conditions for prion amplicons**

**94ºC for 15 min**

**40 cycles of the following:**

94ºC for 20 sec

58ºC for 30 sec^1,2^

72ºC for 60 sec

**72ºC for 3 min**

^1^PCR for DS13a2 is conducted at a primer annealing temperature of 53ºC.

^2^PCR for DS14 is conducted at a primer annealing temperature of 62ºC.

Electrophorese 4 μL of post-PCR samples on an agarose gel and visualize amplicons with ethidium bromide staining.

**PCR cleanup for sequencing.**

For each sequencing reaction, transfer 5.5 μL of post-PCR product to a 96- or 384-well plate containing 7 μL 1:100 Exonuclease I (0.1U/μL).

Incubate in a thermocycler with the following conditions (37ºC 1 hr, 65ºC 20 min, 4ºC hold).

Add 23 μL 100% ETOH, mix, centrifuge 3200 RPM for 30 min at room temp.

Invert upside down and remove suspension. Allow to air dry.

Perform sequence reactions for Applied Biosystems capillary 3730 sequencer (Foster City, CA) using manufacturer reagents and protocol.
