# Supplementary material for: A sequencing strategy for identifying variation throughout the prion gene of BSE-affected cattle
Source: BMC Res Notes. 2008 Jun 23;1:32. doi: 10.1186/1756-0500-1-32 (PMC2525647; doi:10.1186/1756-0500-1-32)
Supplement: Additional file 4 — Supplementary Figure – PRNP PCRs in 96 well format. Illustration of PRNP amplification in a 96 well plate. [file 1756-0500-1-32-S4.ppt]

## Slide 1
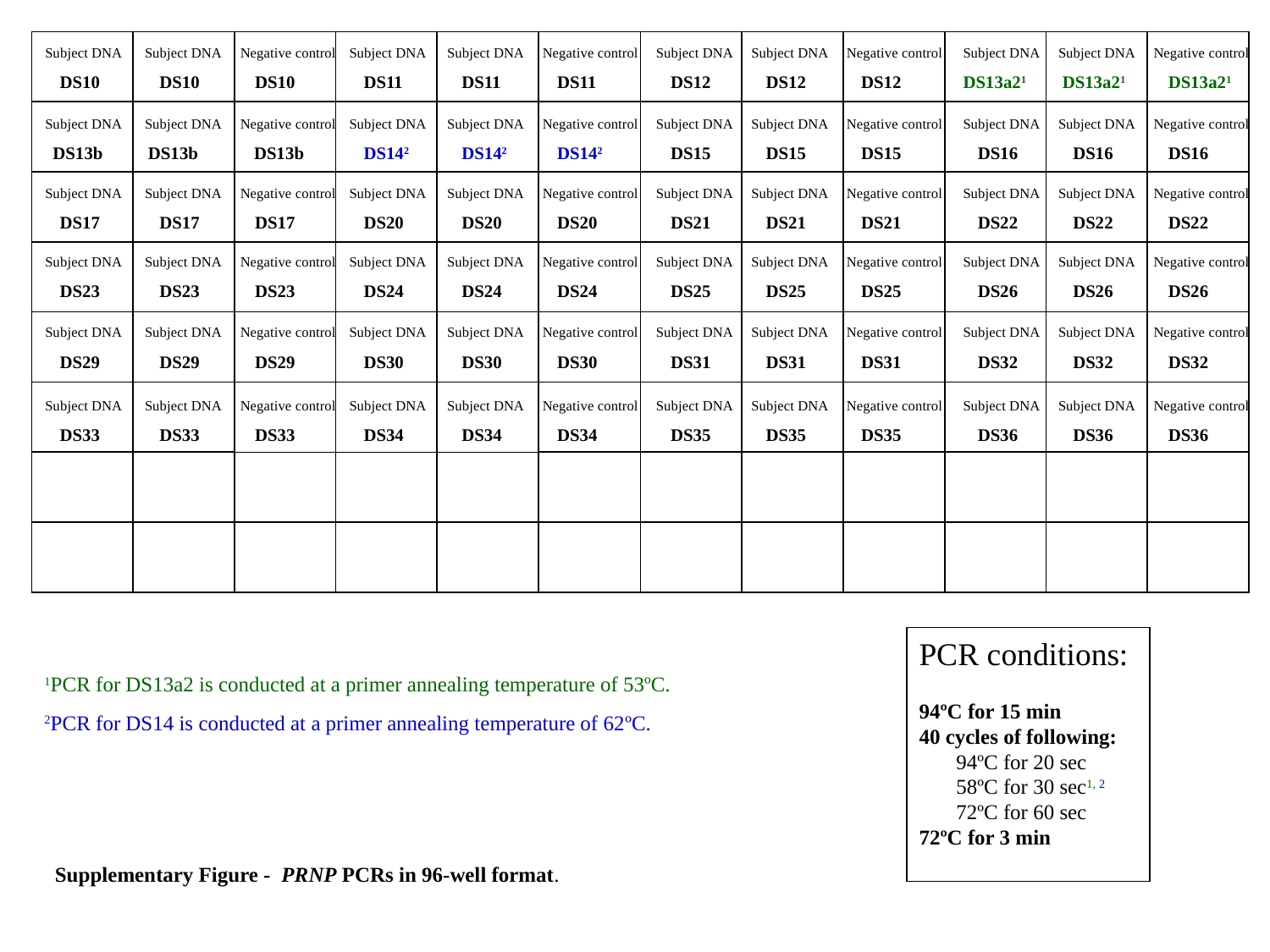

Subject DNA
DS10
Subject DNA
DS10
Negative control
DS10
Subject DNA
DS11
Subject DNA
DS11
Negative control
DS11
Subject DNA
DS12
Subject DNA
DS12
Negative control
DS12
Subject DNA
Subject DNA
Negative control
DS13a21
DS13a21
DS13a21
Subject DNA
Subject DNA
Negative control
Subject DNA
DS142
Subject DNA
DS142
Negative control
DS142
Subject DNA
DS15
Subject DNA
DS15
Negative control
DS15
Subject DNA
DS16
Subject DNA
DS16
Negative control
DS16
DS13b
DS13b
DS13b
Subject DNA
DS17
Subject DNA
DS17
Negative control
DS17
Subject DNA
DS20
Subject DNA
DS20
Negative control
DS20
Subject DNA
DS21
Subject DNA
DS21
Negative control
DS21
Subject DNA
DS22
Subject DNA
DS22
Negative control
DS22
Subject DNA
DS23
Subject DNA
DS23
Negative control
DS23
Subject DNA
DS24
Subject DNA
DS24
Negative control
DS24
Subject DNA
DS25
Subject DNA
DS25
Negative control
DS25
Subject DNA
DS26
Subject DNA
DS26
Negative control
DS26
Subject DNA
DS29
Subject DNA
DS29
Negative control
DS29
Subject DNA
DS30
Subject DNA
DS30
Negative control
DS30
Subject DNA
DS31
Subject DNA
DS31
Negative control
DS31
Subject DNA
DS32
Subject DNA
DS32
Negative control
DS32
Subject DNA
DS33
Subject DNA
DS33
Negative control
DS33
Subject DNA
DS34
Subject DNA
DS34
Negative control
DS34
Subject DNA
DS35
Subject DNA
DS35
Negative control
DS35
Subject DNA
DS36
Subject DNA
DS36
Negative control
DS36
PCR conditions:
94ºC for 15 min
40 cycles of following:
 94ºC for 20 sec
 58ºC for 30 sec1, 2
 72ºC for 60 sec
72ºC for 3 min
1PCR for DS13a2 is conducted at a primer annealing temperature of 53ºC.
2PCR for DS14 is conducted at a primer annealing temperature of 62ºC.
Supplementary Figure - PRNP PCRs in 96-well format.
