# Supplementary material for: A sequencing strategy for identifying variation throughout the prion gene of BSE-affected cattle
Source: BMC Res Notes. 2008 Jun 23;1:32. doi: 10.1186/1756-0500-1-32 (PMC2525647; doi:10.1186/1756-0500-1-32)
Supplement: Additional file 6 — Supplementary Methods – PRNP haplotype reconstructions. Method for determining PRNP haplotypes in the group of 86 Holsteins, the classical BSE case, and her sire. [file 1756-0500-1-32-S6.doc]

**Supplementary Methods - *PRNP* haplotype reconstructions.**

*PRNP* haplotype phase was determined with Phase (version 2.1) [18, 19] using the 19 previously described htSNPs. Manual *PRNP* haplotype reconstruction for the U.S. BSE index case and her sire is demonstrated below.

**Network #1**

Supp. file 7 has a table that shows the nine htSNPs that define *PRNP* haplotypes in Network #1. Both the BSE case and her sire were homozygous for haplotype A (Fig 2, Supp. File 7). Thus, both had homozygous genotypes at all nine htSNPs that define Network #1 and the homozygous haplotype sequence was unambiguous.

BSE case haplotype genotype = TCCGCZGCA

BSE case haplotype allele #1 = TCCGCZGCA (haplotype A, Supp. file 7)

BSE case haplotype allele #2 = TCCGCZGCA (haplotype A, Supp. file 7)

Sire of BSE case haplotype genotype = TCCGCZGCA

Sire of BSE case haplotype allele #1 = TCCGCZGCA (haplotype A, Supp. file 7)

Sire of BSE case haplotype allele #2 = TCCGCZGCA (haplotype A, Supp. file 7)

**Network #2**

Supp. File 7 shows the ten htSNPs that define *PRNP* haplotypes in Network #2. The sire was homozygous for haplotype b (Fig 2, Supp. File 7). Thus, the sire had homozygous genotypes at all ten htSNPs that define Network #2 and the homozygous haplotype sequence was unambiguous.

Sire of BSE case haplotype genotype = ATTGCCGCCC

Sire of BSE case haplotype allele #1 = ATTGCCGCCC (haplotype b, Supp. File 7)

Sire of BSE case haplotype allele #1 = ATTGCCGCCC (haplotype b, Supp. File 7)

The BSE case was homozygous for seven and heterozygous for three of the ten htSNPs that define Network #2.

BSE case haplotype genotype = ATTGCSGCYY

Of the 17 haplotypes listed in Network #2 of Supp. File 7 (haplotypes a-p), only one haplotype combination yields the BSE case genotype.

BSE case haplotype genotype = ATTGCSGCYY

BSE case haplotype allele #1 = ATTGCCGCCC (haplotype b, Supp. File 7)

BSE case haplotype allele #2 = ATTGCGGCTT (haplotype e, Supp. File 7)
